# Improved automated early detection of breast cancer based on high resolution 3D micro-CT microcalcification images

**DOI:** 10.1186/s12885-021-09133-4

**Published:** 2022-02-11

**Authors:** Redona Brahimetaj, Inneke Willekens, Annelien Massart, Ramses Forsyth, Jan Cornelis, Johan De Mey, Bart Jansen

**Affiliations:** 1 Department of Electronics and Informatics (ETRO), Vrije Universiteit Brussel (VUB), Pleinlaan 2, Brussels, B-1050 Belgium; 2 Radiology Department, Universitair Ziekenhuis (UZ) Brussels, Laarbeeklaan 101, Brussels, 1090 Belgium; 3 Pathology Department, Universitair Ziekenhuis (UZ) Brussels, Laarbeeklaan 101, Brussels, 1090 Belgium; 4 IMEC, Kapeldreef 75, Leuven, B-3001 Belgium

**Keywords:** Breast Cancer, Microcalcifications, Computer aided detection and diagnosis systems, X-ray micro-CT, Radiomics, Machine learning

## Abstract

**Background:**

The detection of suspicious microcalcifications on mammography represents one of the earliest signs of a malignant breast tumor. Assessing microcalcifications’ characteristics based on their appearance on 2D breast imaging modalities is in many cases challenging for radiologists. The aims of this study were to: (a) analyse the association of shape and texture properties of breast microcalcifications (extracted by scanning breast tissue with a high resolution 3D scanner) with malignancy, (b) evaluate microcalcifications’ potential to diagnose benign/malignant patients.

**Methods:**

Biopsy samples of 94 female patients with suspicious microcalcifications detected during a mammography, were scanned using a micro-CT scanner at a resolution of 9 *μ*m. Several preprocessing techniques were applied on 3504 extracted microcalcifications. A high amount of radiomic features were extracted in an attempt to capture differences among microcalcifications occurring in benign and malignant lesions. Machine learning algorithms were used to diagnose: (a) individual microcalcifications, (b) samples. For the samples, several methodologies to combine individual microcalcification results into sample results were evaluated.

**Results:**

We could classify individual microcalcifications with 77.32% accuracy, 61.15% sensitivity and 89.76% specificity. At the sample level diagnosis, we achieved an accuracy of 84.04%, sensitivity of 86.27% and specificity of 81.39%.

**Conclusions:**

By studying microcalcifications’ characteristics at a level of details beyond what is currently possible by using conventional breast imaging modalities, our classification results demonstrated a strong association between breast microcalcifications and malignancies. Microcalcification’s texture features extracted in transform domains, have higher discriminating power to classify benign/malignant individual microcalcifications and samples compared to pure shape-features.

## Background

Breast cancer is the most commonly diagnosed cancer in women worldwide counting more than 2 million new cases in 2020 [1]. Early detection and diagnosis of breast cancer is crucial for the overall prognosis and the improvement of the patient’s therapeutic outcome.

Historic evidence related to early indicators of breast cancer, dates back to 1913 when Soloman reported microcalcifications’ (MC) presence in the radiographic examination of a mastectomy specimen [2]. Several decades later (1949), radiologist Leborgne postulated that the presence of MCs may be the only mammographic manifestation of a carcinoma [3]. Ever since first evidence was reported, the role of MCs in the detection of breast cancer has been widely studied.

MCs are present in approximately 55% of all non-palpable breast cancers and responsible for the detection of 85-95% of cases of ductal carcinoma in situ (DCIS) during mammogram scans [4, 5]. However, they are also present in common benign lesions [6] (i.e: breast abnormalities, inflammatory lesions, fibrocystic changes, etc).

Once detected in mammograms, they are categorized according to the Breast Imaging Reporting and Data System (BI-RADS) into typical benign, suspicious and typical malignant. Benign MCs are reported to be larger, round with smooth boundaries; suspicious MCs are reported as coarse heterogeneous, and typical malignant MCs are described as clustered, pleomorphic, fine and with linear branching [7–9].

To date, the chemical composition of breast MCs is categorized into three distinct types: hydroxyapatite (HA), calcium oxalate (CO) and magnesium-substituted hydroxyapatite (Mg-Hap), a special subtype of HA. According to [10], the presence of CO coincided in 81.8% of the cases tested with benign lesions, while HA and Mg-Hap were found in 97.7% of malignant lesions. Further investigation of the chemical composition of MCs is outside of the scope of our paper, but these findings show that there is a physical difference in composition between benign and malignant MCs and hence that it is worth investigating their morphology and texture differences in high contrast 3D images.

Over the years, significant improvements have been achieved regarding breast cancer imaging modalities such us in magnetic resonance imaging (MRI), ultrasound, computed tomography, digital breast tomosynthesis (DBT), etc [11]. Regardless their advantages and disadvantages, mammography still remains the main diagnostic technique. However, the adoption of mammography is not without controversy. As mammography is a projection image, the superposition of tissue can hide MCs or/and alter their appearance depending on their orientation relative to the image plane [12, 13]. Moreover, according to Naseem *et al* [14], 52.2% of the MCs extracted from 937 patients, were absent in mammograms and they were only visible under a histological examination. Hence, mammographic interpretations related to the link between MCs characteristics and malignancy, need to be interpreted with care as their interpretations continue to be a critical element in the on-going efforts to improve the quality of early detection of breast cancer [15,16].

Several computer aided detection and diagnosis (CAD) systems have been developed to assist radiologists to detect and characterise MCs and tumors in different breast imaging modalities. Even though evidence shows promising results [17,18], the current CAD systems involved in clinical or preclinical studies, have still a high number of false positives and false negative rates and so far, MCs characteristics have been mostly studied in 2D or 3D low resolution images.

Since the most accurate and realistic way to determine characteristics of a 3D structure is to use a high resolution 3D imaging technique, attention has been paid to X-ray micro-computed tomography (micro-CT). A relatively small number of studies has focused on high resolution 3D MCs characteristics to detect and diagnose breast cancer [19*–*25].

For the first time, a feasibility on using micro-CT to assess the interior structure of MCs was reported in 2011. The study performed on 16 biopsy samples demonstrated different interior structure patterns of benign and malignant MCs [19].

Willekens et al. [20], were the first to analyze the relationship between 3D shape properties of individual MCs and malignancies. Initially, six 3D shape characteristics of 597 MCs (extracted from 11 samples) were analyzed and it was concluded that MCs belonging to malignant samples, have a more irregular shape compared to benign ones [20]. In a follow-up study on 100 samples, a promising automated sample classification system based only on eight shape and twelve boundary zone features [21] was proposed. A new classification approach (using the same dataset as in [21]) was later on proposed in [22] by clustering MCs based on their shape and texture features.

The relevance of MC’s 3D characteristics as malignancy predictors was further studied in 2017 in 28 samples [23]. Some of their findings were in line with [20], however their structure model index (SMI) was not significantly associated with B-classification of breast lesions. In 2018, the clinical use of MC images generated with high resolution 3D micro-CT scanners was discussed in details by Baran *et al* [24]. Results of this study concluded that high resolution 3D scanners can provide information at a level of details near that of histological images, which would allow much better diagnosis compared to what X-ray imaging modalities allow for.

In our latest work [25], we proposed a CAD system for the characterization of individual MCs. Our classification results confirmed that there is definitely an important link between MCs characteristics and malignancies. A recent study [26], affirmed significant differences between MCs found in malignant and benign canine mammary tumours and their results suggested similarities to MC findings in malignant and benign human breast lesions. Hence, their findings support the further use of this animal model to study human breast cancer.

The main aims of this study were to: (a) explore the feasibility of an automated CAD system that classifies benign and malignant individual MCs and patients based solely on high resolution 3D MCs features and (b) to explicitly contribute to a more accurate understanding of MCs characteristics, the main signs of an early breast cancer. To this end, we perform experiments on a high amount of samples where we: extend our preliminary studies [20–22,25,27,28] by performing more image preprocessing techniques, extracting a higher amount of radiomic features and combining individual MCs results to provide patient diagnosis.

## Materials

### Patients

In this study we have retrospectively included female patients with suspicious MC findings detected during a mammography examination performed between 2007-2012. Subjects underwent minimally invasive vacuum-assisted stereotactic biopsy at the university hospital Brussels (UZ Brussels). Biopsy specimens of 94 women (43 benign and 51 malignant samples), age range 36-83 years and mean subjects age 56.9 ±9.5 years (benign mean age: 57.2 ±9.7, malignant mean age: 56.7 ±9.4) were randomly selected from the UZ Brussels’ breast biopsies archives.

### Breast biopsy

Biopsies were performed with the Mammotome Biopsy System (Ethicon Endo-Surgery, Inc., Johnson & Johnson, Langhorne PA, Pennsylvania, USA) by the department of radiology at UZ Brussels. The extracted samples were stored in blocks of paraffin and they were anatomopathologically examined to obtain the final diagnosis. The tissue samples extracted have a diameter of 3 mm and a length of 23 mm. Further details are explained in [21,27].

### Sample and MCs labeling

During the anatomopathological examination, the pathologist classified samples as malignant or benign depending on whether cancer cells were observed or not. MCs labels were assigned based on the nature of the sample they originated from. As a consequence, it is possible that benign MCs are present in malignant samples [29–31]. However, they were labeled as malignant although their features might indicate benign characteristics. We present in Table 1 an overview of the clinicopathological characteristics for all the involved subjects. In the current study, no clinicopathological information was incorporated in the CAD model.

**Table 1:** Patients’ clinicopathological characteristics. BI-RADS breast density assessment is expressed from A-D scaling: A (<25% glandular), B (25% - 50% glandular), C (51% - 75% glandular, D (>75% glandular). Patient reproductive history is expressed using Gravida-Para (GP) terminology (’has children’ label refers to patient with children but exact number was not specified/saved). The label ’undefined’ indicates cases for which information could not be retrieved from the hospital’ archives or the patient did not provide it

*Characteristics*	*Benign (n=43)*	*Malignant (n=51)*
*Mean age (years ± std)*	57.2 ±9.7	56.7 ±9.4
	A (*n*=4)	A (*n*=8)
	B (*n*=19)	B (*n*=26)
*BI-RADS breast density*	C (*n*=14)	C (*n*=14)
	D (*n*=6)	D (*n*=3)
	No (*n*=40)	No (*n*=44)
*Breast mass*	Yes (*n*=3)	Yes (*n*=7)
	No (*n*=43)	No (*n*=47)
*Distortion*	Yes (*n*=0)	Yes (*n*=4)
	G0P0 (*n*=4)	G0P0 (*n*=3)
	G1P1 (*n*=3)	G1P1 (*n*=8)
	G2P1 (*n*=1)	G2P1 (*n*=2)
	G2P2 (*n*=6)	G2P2 (*n*=8)
	G3P1 (*n*=1)	G3P3 (*n*=3)
	G3P2 (*n*=2)	G4P3 (*n*=1)
*Reproductive history*	G3P3 (*n*=1)	G6P6 (*n*=1)
	G4P3 (*n*=1)	G9P9 (*n*=1)
	G6P6 (*n*=1)	Has children (*n*=2)
	G8P7 (*n*=1)	Undefined (*n*=22)
	Has children (*n*=2)	-
	Undefined (*n*=20)	-
	No (*n*=10)	No (*n*=7)
*Family history with breast cancer*	Yes (*n*=5)	Yes (*n*=7)
	Undefined (*n*=28)	Undefined (*n*=37)
	No (*n*=5)	No (*n*=0)
*Family history with other cancer/s*	Yes (*n*=2)	Yes (*n*=6)
	Undefined (*n*=36)	Undefined (*n*=45)

### Micro-CT imaging

Samples were scanned using a SkyScan 1076 scanner (Brucker microCT, Kontich, Belgium) [32]. The scanner (tube current 167 *μ*A) was composed of a sealed 10-W micro-focus X-ray tube that generated x-rays with a focal spot size of 5 *μ*m. The lower X-ray energies were selected by limiting the spectrum to 60 kV. The X-ray detector (4000 x 2300) consisted of a gadolinium powder scintillator optically coupled with a tapered fiber to a cooled CCD sensor. Further information related to scanner settings can be found in [21,32]. For each sample, projection images were taken every 0.5^∘^ covering a view of 180^∘^ with an exposure time of 1.8 seconds per projection. The total scanning time per sample was 24 minutes. Images were reconstructed using a modified Feldkamp cone-beam algorithm yielding a stack of 2D slices. The 3D sample images have a resolution of 9 *μ*m per voxel and 2291x988x339 voxels.

### Image segmentation

MCs appear on images as regions with higher intensity compared to the local surroundings even though their borders are not always clearly delineated. We used the custom-based segmentation results of [27] as volumes of interests (VOI). The segmentation technique of [27], used six level connected components connectivity to detect connected regions. The connected components with a size smaller than 10 voxels and segments larger than a sphere with a diameter of 1 mm (known as macrocalcifications) were excluded [27]. In total, 3504 MCs were segmented from 94 samples: 1981 MCs from 43 benign samples and 1523 from 51 malignant ones. The mean number of extracted MCs was 46.1 ±58.5 for benign samples and 29.9 ±27.5 for the malignant ones. The image segmentation was performed in Matlab.

### Feature extraction

We extracted a high amount of radiomic features consisting of first order statistical features, shape, texture (Gray Level Co-occurrence Matrix (GLCM), Gray Level Run Length Matrix (GLRLM), Gray Level Size Zone (GLSZM), Gray Level Dependence Matrix (GLDM), Neighbouring Gray Tone Difference Matrix (NGTDM)) and higher order statistical features. Radiomics, aims to quantify phenotypic characteristics on medical images into a high dimensional feature space containing data with high prognostic value [33,34]. In our previous study [25], results were considerably improved when features were computed in Laplacian of Gaussian (LoG) and Wavelet transform domains (area under the curve (AUC) value improved by 11%). Consequently, in this study we extended the amount of image transforms applied.

The applied transform methods are: LoG, three level decomposition of Daubechies Wavelet filters, square, logarithm, squareRoot, exponential and gradient transform. In total, we extracted 2714 features per image. Shape features were extracted only in raw images. The same amount of features per feature class was extracted for all transforms, except for the wavelet transform. For every decomposition level of wavelet filters, features were computed in eight Wavelet subbands (LLL, HLL, LHL, HHL, LLH, HLH, LHH, HHH) as derived by applying a High (H) or Low (L) pass filter in each of the three dimensions. Some wavelet features were removed due to invalid feature values obtained. A summary of all feature classes and the amount of the extracted features per transform method is shown in Table 2. All radiomic feature values were standardized (z-score) prior to classification. Feature extraction was performed on the VOI using PyRadiomics library (version 2.2.0) [35] in Python (version 3.7.3).

**Table 2:** Number of extracted features (extracted on original images and transform domains) per each feature class (shape, first order, GLCM, GLRLM, GLSZM, GLDM, NGTDM)

	*Shape*	*First Order*	*GLCM*	*GLRLM*	*GLSZM*	*GLDM*	*NGTDM*
*Original image*	17	19	24	15	16	14	5
*LoG*	0	19	24	15	16	14	5
*Exponential*							
*Square*							
*Logarithm*							
*Square Root*							
*Gradient Transform*							
*Wavelet*	0	418	528	330	352	308	110

Gray Level Co-occurrence Matrix (GLCM), Gray Level Run Length Matrix (GLRLM), Gray Level Size Zone Matrix (GLSZM), Gray Level Dependence Matrix (GLDM), Neighbouring Gray Tone Difference Matrix (NGTDM), Laplacian of Gaussian (LoG)

### Feature selection

Starting from the high dimensional feature space, we performed feature selection by means of recursive feature elimination (RFE) [36], in order to reduce the risk of overfitting due to the high dimensionality and to achieve our goal to identify a small MCs signature. Chi-squared and fisher score feature selection methods were also explored in our preliminary study [28]. In all the experimental setups, RFE outperformed all the above-mentioned methods. For this reason, in this study we focused only on the RFE method.

RFE is a wrapper feature selection method which selects different subsets of features (to be given as an input for the training of machine learning models) and evaluates their significance based on the classification performance. To select the optimal number of features, for the first 20 features we started with a minimum amount of 2 features to be selected and increment this number with one (aiming to identify a very small number of discriminative features). After the first 20 features tested, we incremented the number of features by 10 until all the extracted features were included. We defined the final best subset of features according to the feature selection frequency among all iterations. In such a way, all the used features were selected on the basis of their stability and relevance.

### Classification

#### Individual MCs classification

The performance of four classification algorithms was investigated: Random Forest (RF), Support Vector Machine (SVM), Multilayer Perceptron (MLP) and AdaBoost. Experiments were performed using leave-one-subject-out cross validation. Every experiment was repeated 30 times on shuffled data to ensure the stability of results. When SVM and AdaBoost algorithms are used, results among multiple iterations are the same as there is no stochasticity in the methods, nor are they influenced by training data order. Models’ performances were measured in terms of accuracy, sensitivity, specificity, AUC and F-score. All implementations of the classification algorithms and RFE were done in Python (version 3.7.3) using ScikitLearn (version 0.21.2).

#### Sample classification

One of the clinical goals, is the possibility to establish diagnosis at a patient level. Therefore, we investigated:

*A thresholding approach* - if the number of malignant MCs predictions for a given sample exceeded a specified threshold value, the sample was considered to be malignant (i.e: if the number of the predicted malignant MCs of a sample was larger than 20% of the entire sample MCs, the sample was classified as malignant). The threshold values evaluated start from 5% up to 50%, incremented by 5. We adopted this approach, because it is practically impossible to establish a ground truth label for each MC, while for a sample this is perfectly feasible.

*Multiple instance-learning (MIL) algorithms* - the general assumption of MIL algorithms is that every positive bag (i.e. sample) contains at least one positive instance (i.e. malignant MC) while negative bags contain only negative instances (positive/negative refers to malignant/benign and bag/instance refers to sample/MC respectively). We considered suitable the use of MIL algorithms for sample classification given the ambiguity in MCs inheriting sample labels. The algorithms used are: normalized set kernel (NSK), statistics kernel (STK), sparse multiple instance learning (sMIL), maximum bag margin SVM (MISVM), maximum pattern margin SVM (miSVM), multi instance learning by semi-supervised SVM (MissSVM) [37,38]. Different MIL algorithms make different assumptions about positive instances present in samples as explained in details in [37,38]. All the resulting representations were used to train a base SVM classifier. In terms of feature selection, we test the performance of the MIL algorithms starting from 5 up to 300 best features (as derived from RFE), incremented by 10.

## Results

### Results of individual mCs classification

Results of individual MCs classification experiments for the four aforementioned classifiers (with/without feature selection) are shown in Tables 3 and 4. We initially calculated accuracy, sensitivity, specificity, AUCs and F-score values for every classifier and iteration separately. Results reported in Tables 3 and 4, represent the average and standard deviation (std) of these metrics among the 30 repetitions for each classifier. When using all the extracted features, we reached an accuracy of 77.03%, sensitivity of 60.46%, specificity of 89.77%, F-score of 76.35% and AUC value of 80.10% with RF classifier.

**Table 3:** Results (expressed in percentage) of individual MCs classification experiments among 30 repetitions, no feature selection method applied

*Classifier*	*Accuracy*	*Sensitivity*	*Specificity*	*AUC*	*F score*
MLP	71.79 ±1.05	64.65 ±1.21	77.28 ±1.34	77.16 ±0.93	71.68 ±0.01
**RF**	**77.03** ±**0.13**	**60.46** ±**0.21**	**89.77** ±**0.15**	**80.10** ±**0.06**	**76.35** ±**0.01**
SVM	73.80 ±0.0	61.39 ±0.0	83.34 ±0.0	77.87 ±0.0	73.39 ±0.0
AdaBoost	75.68 ±0.0	61.58 ±0.0	86.52 ±0.0	77.89 ±0.0	75.17 ±0.0

Area Under the Curve (AUC), Multi Layer Perceptron (MLP), Random Forest (RF), Support Vector Machine (SVM)

**Table 4:** Results (expressed in percentage) of individual MCs classification experiments among 30 repetitions, RFE feature selection method applied

	*Accuracy*	*Sensitivity*	*Specificity*	*AUC*	*F score*	*Features*
MLP	75.46 ±0.65	63.44 ±1.20	84.70 ±0.74	80.57 ±0.58	75.08 ±0.01	300
**RF**	**77.32** ±**0.09**	**61.15** ±**0.16**	**89.76** ±**0.14**	**81.18** ±**0.04**	**76.67** ±**0.01**	**300**
SVM	75.74 ±0.0	60.93 ±0.0	87.12 ±0.0	78.24 ±0.0	75.17 ±0.0	80
AdaBoost	76.42 ±0.0	63.09 ±0.0	86.67 ±0.0	77.40 ±0.0	75.97 ±0.0	300

Area Under the Curve (AUC), Multi Layer Perceptron (MLP), Random Forest (RF), Support Vector Machine (SVM)

**Fig. 1:**
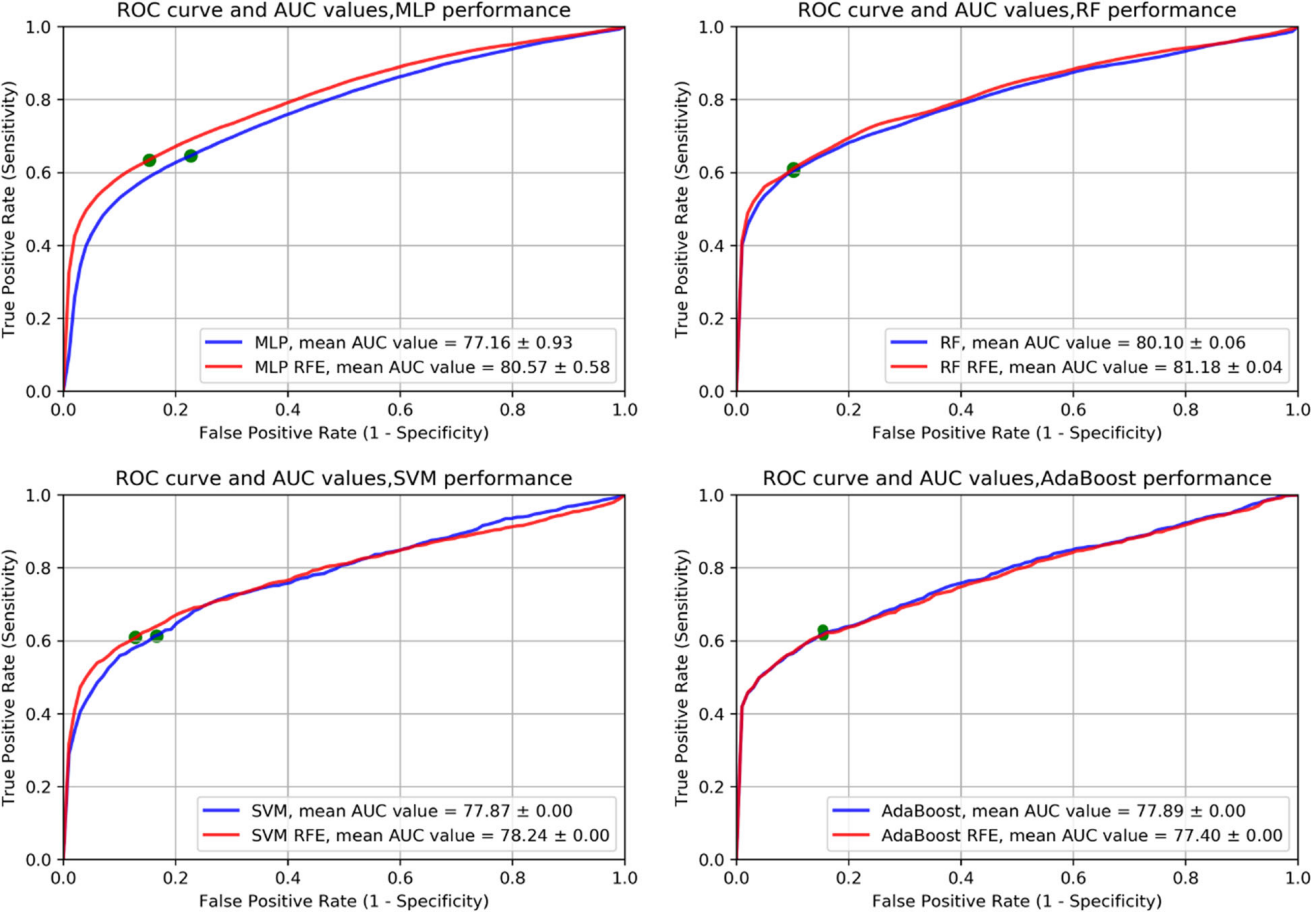
ROC curves and AUC values corresponding to experimental results reported in Tables 3, 4. The green points represent the decision threshold for the reported results in the corresponding tables

When RFE feature selection was applied, an accuracy of 77.32% ±0.09, sensitivity of 61.15% ±0.16, specificity 89.76% ±0.14, F-score 76.67% ±0.01 and AUC 81.18% ±0.04 were obtained with the RF classifier using 300 features (see Table 4). All AUC values improved (except the AdaBoost AUC value) when we performed RFE feature selection method (see also Fig. 1). A paired t-test was used to analyze whether feature selection had a significant influence on the classification performance (tested on AUC values). At a p value <0.05, we got significantly different results for both MLP and RF when performing feature selection. For SVM and AdaBoost, no statistical significant difference could be computed since there are no differences among the 30 repetitions.

To determine the diagnostic performance of the classification algorithms, we focus on AUC values. Among the 30 repetitions, RF showed the best performance: AUC of 81.18% ±0.04. Among the features that were mostly selected, for the best classification result obtained (RF, 300 features), 87 features belonged to first level wavelet decomposition, 44 second level decomposition wavelet, 64 third level wavelet decomposition, only 1 shape related feature, 40 exponential, 10 gradient, 14 LoG, 5 logarithm features and 7 texture features extracted on original images.

### Results at sample level

Sample level results for the different classifiers and threshold values tested (with/without feature selection) are shown in Tables 5 and 6. They are calculated as follows: for a given sample, we group all its individual MC predictions over the 30 repetitions (same predictions as outputted from MCs classification experimental-setups described above) and we apply the different threshold values mentioned over the grouped predictions; if the number of malignant-predicted MCs exceeds the threshold value, we labeled the sample as malignant, otherwise as benign. We computed sensitivity, specificity, F-score and accuracy on these re-labeled patients whereas the individual sample accuracy is defined as 100% if the assigned label matches with the sample ground-truth label, else 0%. The accuracy reported is calculated as the average of 94 sample accuracies per classifier tested. AUCs values can not be computed for sample classification as we do not have classification probability prediction values per sample.

**Table 5:** Sample classification, thresholding approach results (expressed in percentage), no feature selection

	*No Feature Selection*				
*Threshold*	*Classifier*	*Accuracy*	*Sensitivity*	*Specificity*	*F score*
50%	MLP	78.72 ±41.15	70.59	88.37	78.67
45%	SVM	78.72 ±41.15	74.51	83.72	78.75
**40**%	**MLP**	**80.85** ±**39.56**	**80.39**	**81.39**	**80.87**
35%	AdaBoost	79.79 ±40.37	72.55	88.37	79.77
35%	MLP	78.72 ±41.15	80.39	76.74	78.72
30%	SVM	78.72 ±41.15	76.47	81.36	78.76
30%	AdaBoost	77.66 ±41.88	74.51	81.40	77.70
25%	RF	80.85 ±39.56	76.47	86.04	80.88
25%	AdaBoost	78.72 ±41.15	80.39	76.74	78.72
20%	RF	78.72 ±41.15	76.47	81.39	78.76
15%	RF	78.72 ±41.15	82.35	74.41	78.67
10%	RF	75.53 ±43.22	86.27	62.79	75.09
10%	AdaBoost	71.28 ±45.49	92.16	46.51	69.46
5%	RF	74.47 ±43.84	96.07	48.83	72.69

Multi Layer Perceptron (MLP), Random Forest (RF), Support Vector Machine (SVM)

**Table 6:** Sample classification, thresholding approach results (expressed in percentage), RFE feature selection

	*Feature Selection*				
*Threshold*	*Classifier*	*Accuracy*	*Sensitivity*	*Specificity*	*F score*
50%	MLP	76.6 ±42.57	64.70	90.69	76.37
45%	MLP	77.66 ±41.88	68.62	88.37	77.58
40%	AdaBoost	79.79 ±40.37	70.59	90.70	79.71
35%	AdaBoost	80.85 ±39.56	74.51	88.37	80.85
35%	SVM	78.72 ±41.15	70.58	88.37	78.67
30%	AdaBoost	80.85 ±39.56	78.43	83.72	80.89
30%	SVM	78.72 ±41.15	72.55	86.05	78.72
**25**%	**AdaBoost**	**84.04** ±**36.82**	**86.27**	**81.39**	**84.03**
25%	RF	78.72 ±41.15	76.47	81.39	78.76
20%	AdaBoost	80.85 ±39.56	88.24	72.09	80.66
15%	RF	77.66 ±41.88	82.35	72.09	77.57
10%	RF	75.53 ±43.22	86.27	62.79	75.09
10%	SVM	74.47 ±43.84	88.24	58.14	73.74
5%	RF	73.4 ±44.42	92.15	51.16	72.03

Multi Layer Perceptron (MLP), Random Forest (RF), Support Vector Machine (SVM)

Some of the best results obtained are shown in Tables 5 and 6. We obtained an accuracy of 80.85% ±39.56, sensitivity of 80.39%, specificity of 81.39% and F-score of 80.87% for a 40% threshold value using MLP classifier (Table 5). When applying RFE and using a 25% threshold value, we were able to reach higher results and predict samples with 84.04% ±36.82 accuracy, 86.27% sensitivity, 81.39% specificity and 84.03% F-score, using AdaBoost classifier (Table 6).

**Fig. 2:**
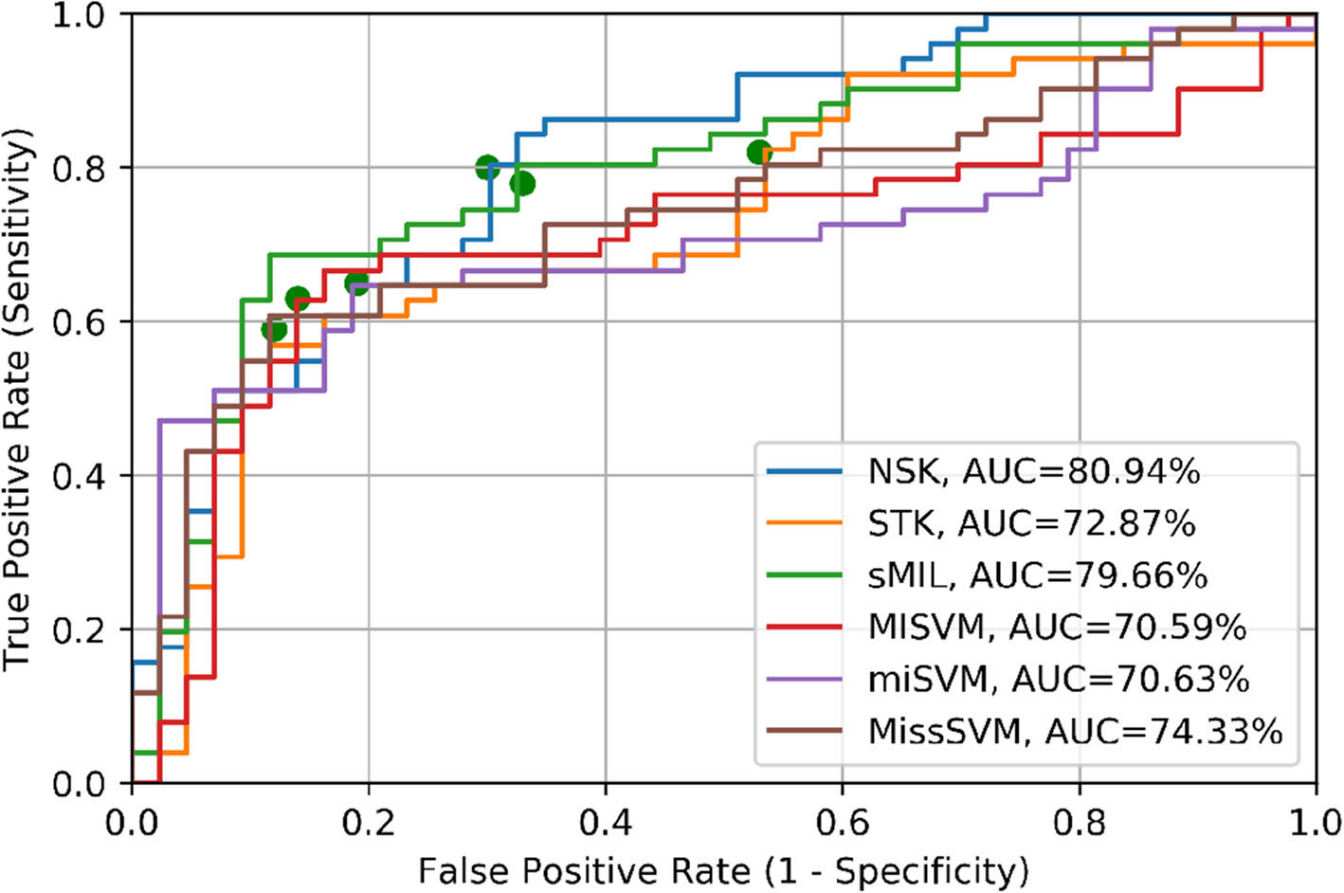
ROC curves and AUC values, multiple instance-learning algorithms. The green points represent the decision threshold for the reported results in the Table 7

By using multiple instance-learning algorithms, we clas- sified samples with an accuracy of 75.53%, sensitivity 80.39%, specificity 69.76%, F-score 75.44% and AUC value of 80.94% with a NSK classifier (150 features). Results are shown in Table 7 and ROC curves (computed on 94 sample probability predictions) in Fig. 2.

**Table 7:** Sample classification, multiple instance-learning algorithms results (expressed in percentage)

	*Accuracy*	*Sensitivity*	*Specificity*	*AUC*	*F score*	*Nr of features*
**NSK**	**75.53**	**80.39**	**69.76**	**80.94**	**75.44**	150
STK	65.95	82.35	46.51	72.87	64.70	150
sMIL	73.40	78.43	67.44	79.66	73.30	50
MISVM	73.40	62.75	86.04	70.59	73.21	150
miSVM	72.34	64.70	81.39	70.63	72.28	150
MissSVM	72.34	58.82	88.37	74.33	71.93	30

Area Under the Curve (AUC), Normalized set kernel (NSK), Statistics kernel (STK), Sparse multiple instance learning (sMIL), Maximum bag margin support vector machine (MISVM), Maximum pattern margin support vector machine (miSVM), Multi instance learning by semi-supervised support vector machine (MissSVM)

## Discussion

In this study, we extend our latest work [25] by: (a) exploring more image transform methods, (b) extracting a higher amount of radiomic features, (c) optimising feature extraction, feature selection and classification parameters, (d) evaluating the performance of more machine learning algorithms to classify individual MCs, (e) analysing and evaluating the relevance of individual MCs to provide breast cancer diagnosis at a sample level using a voting scheme methodology and multiple instance-learning classification algorithms, (i) providing robustness of our results.

We outperform 1 the individual MCs results reported in our previous work [25]: accuracy 75.88%, sensitivity 62.13%, specificity 86.39% and AUC 77.03%. In the current paper we obtain an accuracy of 77.32%, sensitivity of 61.15%, specificity of 89.76%, AUC of 81.18%. We also outperform the sample classification results reported by Temmermans et al. (in [21]): accuracy 70%, sensitivity 98%, specificity 40% with the following figures of merit: accuracy of 84.04%, sensitivity of 86.27%, specificity of 81.39%).

Among all the preprocessing steps performed, the image transform methods applied and parameter’s optimization, had a significant contribution to achieve the above-mentioned results. The robustness and reliability of our system are confirmed by: (a) the low standard deviation values obtained for all the reported metrics during the individual MCs classification repeated trials, (b) the consistency of results over different machine learning algorithms.

All the individual MCs extracted from a sample inherit the sample label. Therefore, it is not feasible to obtain results close to 100% for the individual MCs classification because benign MCs may occur in malignant samples. Moreover, biopsy samples were only collected because the radiologist found suspicious signs on the mammogram, which causes a significant bias in all the collected data. Nevertheless, our ability to discriminate so accurately between suspicious MCs (present in benign lesions) and malignant ones, confirms that there is a clear difference between MCs originating from malignant and benign lesions.

The association between MCs and breast malignancies has already been stated for decades by studying MCs properties on 2D projections or low resolution 3D images. It is worth stating that the effectiveness of conventional breast imaging modalities to diagnose breast cancer based only on MCs properties provided they are combined with other clinical examinations, is widely accepted (approaching nearly 100% sensitivity and specificity [39]). MCs show high contrast on mammograms, and more and more claims related to the properties of MCs are made based *only* on observations of 2D mammography images. The fact that even by studying MC characteristics in high resolution 3D images, we still encounter difficulties to characterize malignancy indicates that current 2D mammography analysis of MCs should be used cautiously.

The classification of individual MCs served as an intermediate step towards our ultimate goal of performing patient classification. We assessed several threshold values on the amount of MCs classified as malignant to provide patient diagnosis solely based on MCs properties. Our proposed thresholding approach for patient classification, tends to overcome the fact that we deal with a so-called weakly supervised classification problem because the ground truth for individual MCs is not available and only the ground truth of complete samples is known.

Despite the fact that in clinics one malignant MCs should classify the entire sample as malignant, benign MCs may coexist in a malignant sample. Therefore, to avoid miss-classifying the entire sample because of some miss-classified instances, a high AUC-threshold value is appropriate, namely up to 25% as the one we obtained in Table 6.

The high std values obtained in all sample classification experiments should be interpreted with caution. As explained, if the final sample prediction matches with its corresponding ground-truth label, the sample accuracy is 100% otherwise 0%. Even with only one miss-classified sample, the std value would still be more than 10% due to fact that it is calculated over these two extreme accuracy values.

Performance results obtained by using MIL algorithms, were lower than expected. Despite considerable efforts, we managed to only classify correctly 75.53% of our samples using the NSK classifier. Ideally, MIL algorithm should have yielded superior classification performance compared to the manual thresholding approach. A potential reason might be that in all the algorithms tested, the classifier used to classify bags (i.e. samples) is SVM. The performance of SVM in Tables 5 and 6, also shows that SVM does not perform better compared to other classifiers used (i.e: MLP, RF, AdaBoost). A combination of the used MIL algorithms but tested with other base classifiers, would probably result in similar or higher results. Moreover, it is unclear how well MIL algorithms’ assumptions match the real distribution of malignant MCs in malignant samples.

There exists only one other study that has directly evaluated the relevance of 3D MCs structures as a predictor of malignancy [23]. In their evaluation, they analyse in all lesion groups (classified according to the B-classification system) the number, volume, SMI and morphology of suspicious non-monomorphic (fine linear, fine pleomorphic, coarse heterogeneous) MCs. Their findings show that the shape (based on the SMI) of MCs is not significantly associated with the B-classification of breast lesions. Even though we follow different classification approaches and perform experiments at a larger scale on almost a three times larger dataset, our findings confirm that pure shape features are not the most significant features to capture differences among MCs found in benign and malignant lesions.

Despite the fact that in other similar studies shape features extracted from 2D or 3D images have almost always been reported among the most important selected features [20,27,40], we found that high order texture features are ranked higher in terms of feature importance. Only one shape feature (elongation) was selected during the feature selection process (chosen as the 205th most important one) whereas, texture features extracted in transform domains (mainly in wavelet domain) have the most significant predictive power in our classification model. Their potential to be used as an important tool for MCs classification has already been argued for many years [36,41,42] and also proven in our preliminary study [25].

The results achieved are relevant for several potential application scenarios. One such scenario is to provide (almost) real-time diagnosis immediately after extracting the biopsy sample. A multitude of studies have reported that patients experience high levels of anxiety and depression while waiting to obtain their breast biopsy results [43–45]. Although largely unstudied, a few patient surveys exist on current practice versus patient preference with respect to the disclosure of biopsy results [46,47]. According to Attai et al., 82% of breast cancer patients who received their cancer diagnosis two days after the initial biopsy, would have preferred to receive their results in a shorter wait time [46]. Usually, patients have to wait around one week to get their biopsy results. This waiting period, besides the significant economical costs for the healthcare system, considerably impacts the mental state of the patient. Even though this is a rough estimation, with our system patient diagnosis can be provided within the next 30 minutes from the tissue extraction process (including scanning time, loading the large image volume, performing all pre-processing steps and classifying samples).

If in-vivo high resolution 3D screening would be possible, our results have the potential to be translated into clinical practise immediately. Under this assumption, as soon as the radiologists would suggest that the patient should do a screening examination, the breast can be screened in vivo and combined with radiologist assessment [48], the system could be used to provide benign/malignant diagnosis immediately. Two direct benefits would be: (a) a considerable reduction on the number of unnecessary biopsies that turn out to be benign, (b) the possibility for early detection of the tumor before it has aggravated. Important to emphasise is that early diagnosis is vitally important to develop an effective treatment strategy.

The usage of micro-CT scanners to provide (near) real time diagnosis has already been discussed (yet not applied for MCs). Evidence shows that 15%-35% of patients who already had a first breast tumor removal, undergo a second re-excision procedure because of positive pathological boundaries [49]. Imaging of intraoperative surgical specimens for breast tumor boundary assessment in real time, has already been evaluated [50,51] and proven to provide diagnostic images with near histological levels of detail. As already argued, significant positive impacts could be obtained by using micro-CT in this diagnostic system.

If in the near future, prior to the final histopathological examinations, biopsy samples would be routinely scanned with a high resolution 3D scanner, the resulted MC images collected, could impart high value information. The creation of publicly available databases with high resolution 3D MCs images (currently none existing), is essential to further extend the knowledge on MCs diagnostic power.

To implement all the scenarios discussed in clinical practise, a sensitivity much closer to 100% (such that malignant samples will not be missed) should be pursued while maintaining a high balance between accuracy and specificity. Given the fact that convolutional neural networks (CNNs) have already proven to outperform breast cancer systems focused only on hand-crafted radiomic features [18], we strongly believe that in our future work we will be able to improve upon current state of the art by using deep CNN architectures, if sufficient data would be available.

However, 3D high resolution breast imaging in-vivo is not expected to become available in the near future. Despite the considerable advances over the last years (i.e: higher image resolution, more efficient reconstruction algorithms etc), the main limitation of micro-CT remains the high amount of radiation dose that it would transmit to the patient. Even though a trade-off between the radiation dose and the desired image quality can be made, still it is at unacceptable levels as it may induce cancer to the patient [52,53]. The exponentially growing number of studies focusing on micro-CT scanners, underpins the increased importance of this imaging modality and the ongoing optimization efforts to provide in-vivo high resolution scanning [11,51,52].

With our findings we want to convey several messages: (1) using micro-CT imaging to evaluate 3D MCs structures at a micrometer scale can potentially help clinicians in the early detection, diagnosis, treatment and management of breast cancer, (2) the potential of radiomic features (to reveal important image characteristics) and of machine learning algorithms (to classify images) can considerably reduce costs for the healthcare system and avoid unnecessary physical interventions and their psychological consequences, (3) with our proposed system, we intend to help other studies to pave the way towards more effective CAD systems, especially to those making claims based only on mammographic MCs analysis, (4) further improvements on the current limitations of micro-CT will have an enormous impact not only on early diagnosis but also on personalized treatment evaluations, (5) our results support the idea that more thorough analysis of high resolution 3D MCs structures will reveal significant currently-hidden information about MCs diagnostic value.

In our future work, we aim to pursue higher sensitivity while maintaining a good balance between all the classification metrics reported. Towards this goal, we intend to evaluate deep learning algorithms, semi-supervised classification methods and to artificially enlarge our dataset using augmentation techniques. As a long-term goal, we envisage to perform in depth studies to: find correlations between high resolution 3D MCs structures and the different tumor types; test the association between MCs features and clinicopathological/mammogram characteristics; evaluate if adding such features will increase the CAD model performance.

## Study limitations

Our study has several limitations. (a) Our main limitation is the fact that there is a ground truth for samples but not for individual MCs. Benign MCs, potentially present in a malign sample are labeled malignant in our training data, while their feature values may indicate typical benign properties. As a consequence, our training process might be influenced. To tackle this limitation, ideally we would need to isolate benign MCs in the specimen that have both benign and malignant ones. However, to the best of our knowledge, it is nearly impossible to achieve this due to tissue distortion issues.

(b) We have included in our study only MCs from patients with suspicious findings on their mammograms. We firmly believe that significantly better results can be achieved by including in the training set also MCs present in typical benign samples.

In real practise, it might be very difficult to find healthy females that accept to undergo a biopsy with the sole purpose of studying their MCs findings. As a consequence, we believe that we will always be dealing with the most suspicious cases to diagnose in our trial system.

(c) Despite the fact that we are conducting research on the highest number of 3D high resolution MCs images ever reported, we can not assume that we have: enough samples and a perfectly balanced dataset (43 benign samples:1981 MCs vs 51 malignant samples:1523 MCs). Furthermore, for a few samples, we are making predictions based on a very low number (1-5) of MCs extracted. While data augmentation and application of different augmentation ratios may be considered as a potential solution, we hope that the current results obtained will provide further financial support/s to pursue studies on a larger sample size.

(d) Samples included in the study were collected up to 10 years ago. Far less lab results were routinely collected at the time, compared to nowadays. This made it impossible to correlate our findings to certain tumour markers.

(e) The biopsy samples were scanned in 2013 with a micro-CT scanner offering a resolution of 9 *μ*m. Nowadays, the resolution of a micro-CT scanner can reach up to 1 *μ*m [54].

## Conclusion

Our study is the largest one evaluating the feasibility of developing a CAD system that provides breast cancer diagnosis based solely on MC features extracted from high resolution 3D images.

After several preprocessing techniques applied, we achieved state of the art results in diagnosing benign/malignant MCs instances and entire samples by studying MCs characteristics at a level of details beyond what is currently possible by using other conventional breast screening modalities.

Except from proving a strong association between image features of MCs and breast malignancies, we further expand the boundaries of already-known knowledge by concluding that when studying high resolution 3D MCs structures, texture features extracted in transform domains have higher predictive power to distinguish MCs present in malignant lesions than pure shape features.

## Data Availability

The data generated and/or analyzed during the current study are not publicly available. However, the features extracted are available from the corresponding author on reasonable request.

## Abbreviations

RF: Random forest
HA: Hydroxyapatite
CO: Calcium oxalate
STK: Statistics kernel
MC: Microcalcification
VOI: Volume of interest
STD: Standard deviation
UZ: University hospital
MCs: Microcalcifications
AUC: Area under the curve
NSK: Normalized set kernel
SMI: Structure model index
LoG: Laplacian of Gaussian
MLP: Multi layer perceptron
GLSZM: Gray Level Size Zone
SVM: Support vector machine
DCIS: Ductal carcinoma in situ
MIL: Multiple instance-learning
MRI: Magnetic resonance imaging
DBT: Digital breast tomosynthesis
CNN: Convolutional neural network
RFE: Recursive feature elimination
sMIL: Sparse multiple instance learning
GLDM: Gray Level Dependence Matrix
Micro-CT: Micro-computed tomography
GLRLM: Gray Level Run Length Matrix
HER2: Human epidermal receptor type 2
GLCM: Gray Level Co-occurrence Matrix
CAD: Computer aided detection and diagnosis
Mg-Hap: Magnesium-substituted hydroxyapatite
NGTDM: Neighbouring Gray Tone Difference Matrix
BI-RADS: Breast imaging reporting and data system
MISVM: Maximum bag margin support vector machine
miSVM: Maximum pattern margin support vector machine
MissSVM: Multi instance learning by semi-supervised support vector machine

## Footnotes

1 This footnote further illustrates that we really outperform the results of [25]. If we select on the AUC curve in [25] a sensitivity value of 61.15%, which is the value reported in the current paper, then the corresponding performance values in [25] are: specificity 85.54%, accuracy 74.96%, which are all smaller than the values obtained in the current paper. If we select on the AUC curve of the current paper the sensitivity reported in [25], namely 62,13%, we obtain a specificity of 86.5% and an accuracy of 76.35%, which are all higher than the values reported in [25].
